# Pregabalin-associated Discontinuation Symptoms: A Case Report

**DOI:** 10.7759/cureus.3425

**Published:** 2018-10-08

**Authors:** Sadiq Naveed, Amber E Faquih, Amna Mohyud Din Chaudhary

**Affiliations:** 1 Psychiatry, KVC Prairie Ridge Psychiatric Hospital, Kansas City, USA; 2 Psychiatry, Dow University of Health Sciences, Karachi, PAK; 3 Psychiatry, Nishtar Medical College & Hospital, Multan, PAK

**Keywords:** pregabalin, discontinuation, adverse effect

## Abstract

Pregabalin is used for the treatment of neuropathic pain, partial seizures, generalized anxiety disorder, social anxiety disorder, and insomnia. The discontinuation symptoms of pregabalin are mild to moderate which resolve in about one week after the discontinuation of pregabalin. This case report describes the withdrawal symptoms in a 62-year-old patient despite a slow titration over a period of several weeks. It highlights the importance of cautious monitoring of withdrawal symptoms during the period of taper.

## Introduction

Pregabalin is used for neuropathy pain and as an adjunctive therapy for partial seizures [1-2]. It is also used off-label as an adjunct for the treatment of generalized anxiety (GAD), social anxiety disorder, and insomnia in fibromyalgia [3]. Pregabalin exerts its anxiolytic effects through its high-affinity binding to the alpha-2-delta sub-unit of the P/Q type voltage-gated calcium channels in “over-excited” presynaptic neurons, thereby reducing the release of excitatory neurotransmitters [4]. The mechanism of action does not appear to be mediated through effects on gamma-aminobutyric acid (GABA) despite its molecular resemblance to GABA [4]. Pregabalin is completely absorbed when taken orally with linear dose-absorption relationship [4]. It is eliminated via the kidneys with minimal metabolism and has no significant drug-drug interactions [4]. The pregabalin has a half-life of 5.5-6.7 hours, independent of dose and repeated dose administration [1]. The mild to moderate transient dose-related adverse effects of pregabalin include dizziness, somnolence, and peripheral edema [4]. The discontinuation symptoms of pregabalin have been reported in few case reports which happen within one week of discontinuation.

## Case presentation

**Figure 1:**
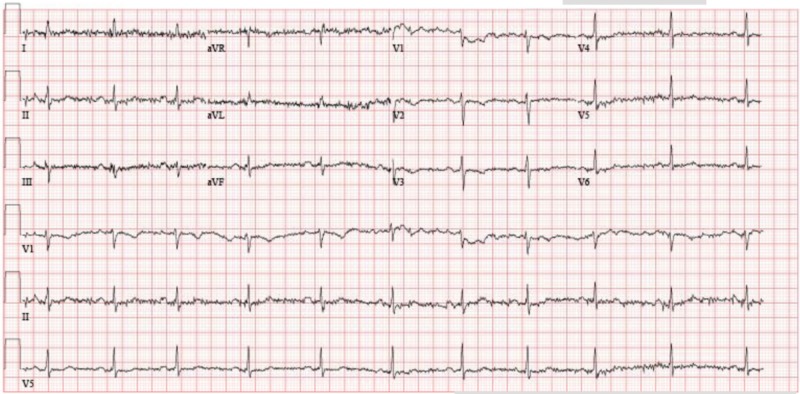
Image of electrocardiogram. Ventricular rate 64 beats per minute PR interval        188 milliseconds QRS duration      80 milliseconds QT/QTc     424/437 milliseconds P-R-T axes   65 9     42 Normal sinus rhythm Nonspecific ST and T wave abnormality Abnormal EKG When compared with EKG of June 20, 2018 ST less depressed in inferior leads T wave inversion no longer evident in inferior leaders Nonspecific T wave abnormality no longer evident in lateral leads

A 62-year-old white female with a known history of rheumatoid arthritis, pancreatitis, migraine, fibromyalgia, cervical disc diseases, asthma, general anxiety disorder, and unspecified depressive disorder was admitted to hospital with complaints of chest pain, extreme weakness of legs, diffuse body tremors, aches, worsening of anxiety, insomnia, and increased fearfulness. She described her chest pain as "floating" from the left to the right side with radiation into her right arm, jaw, and right upper back associated with occasional dyspnea, palpitations, and dizziness. These symptoms started after the patient was going through the pregabalin taper. She also reported suicidal ideations at the night of admission. She was given after an intravenous push of lorazepam 1 mg to help with anxiety and was admitted to telemetry unit. Cardiology and psychiatry were consulted. On cardiology consultation, all cardiogenic causes of chest pain were excluded, and electrocardiogram (EKG), cardiac enzymes, and positron emission tomography-myocardial perfusion imaging were normal. The initial EKG was of poor quality and it was repeated. The repeat EKG was done, and the cardiac causes for the chest pain were ruled out (Figure 1).

During her visit with the psychiatrist, she denied any suicidal ideations and reported that this extremely worsening of anxiety, fearfulness, and somatic symptoms started after pregabalin taper was initiated about three months ago. Pregabalin taper was initiated by her outpatient rheumatologist because she was concerned about the long-term effects of pregabalin on brain waves. She was previously taking pregabalin 150 mg three times a day which was tapered at 150 mg intervals every month. In the last month of pregabalin taper when the dose was 150 mg daily, she started to have discontinuation symptoms which she described as “feeling of going crazy.” Bupropion extended-release (XL) 150 mg in the morning was started in outpatient a week before admission to address possible worsening of anxiety and depression based on the past history of a favorable response. She was already taking sertraline 100 mg in the morning, trazodone 100 mg at night, and alprazolam 0.5 mg three times a day. Her bupropion XL was discontinued considering its adverse effect on anxiety and lack of evidence for anxiety disorders. Alprazolam 0.5 mg was increased to every six hours as needed with a recommendation of considering duloxetine in future to address both anxiety and fibromyalgia. Subsequently, the patient continued to report an improvement in her chest pain, somatic symptoms, anxiety, and diffuse body tremors. Her symptoms eventually resolved and the patient was discharged. She was advised to schedule a follow-up visit with her outpatient physicians.

## Discussion

The existing knowledge about the discontinuation of pregabalin is limited. However, the existing case studies suggest diaphoresis, tachycardia, hypertension, tremors, diarrhea, agitation, paranoia, auditory hallucinations, mutism, self-mutilation, and suicidal attempt as common symptoms of pregabalin discontinuation [5-8]. It is hypothesized that the mechanism of withdrawal symptoms of pregabalin is similar to benzodiazepine and ethanol, where discontinuation increases the production of the enzyme responsible for producing GABA [7]. Pregabalin is a schedule V drug due to its potential for abuse [7]. Pregabalin taper has been associated with an increased risk of delirium and confusion [7]. The discontinuation of pregabalin is considered to be clinically safe if it was tapered gradually over a period of one week [5-7]. In our patient, the discontinuation symptoms were observed even after a slow taper over a period of several weeks, warranting a careful assessment of possible discontinuation symptoms.

## Conclusions

This case report presents the late onset of discontinuation symptoms of pregabalin. Usually, these symptoms resolve in one week after pregabalin taper. However, our patient continued to have these symptoms for several weeks. It highlights the importance of cautious monitoring and gradual titration in patients, taking pregabalin for a long time.
